# CD39 in the development and progression of pulmonary arterial hypertension

**DOI:** 10.1007/s11302-022-09889-9

**Published:** 2022-08-10

**Authors:** Abbey Willcox, Natasha Ting Lee, Harshal H. Nandurkar, Maithili Sashindranath

**Affiliations:** Present Address: Australian Centre for Blood Diseases, Central Clinical School, Monash University and Alfred Health, Monash AMREP Building, Level 1, Walkway, via The Alfred Centre, 99 Commercial Road, Melbourne, VIC 3004 Australia

**Keywords:** Pulmonary arterial hypertension, Endothelial dysfunction, CD39

## Abstract

Pulmonary arterial hypertension (PAH) is a devastating progressive disease characterised by pulmonary arterial vasoconstriction and vascular remodelling. Endothelial dysfunction has emerged as a contributing factor in the development of PAH. However, despite progress in the understanding of the pathophysiology of this disease, current therapies fail to impact upon long-term outcomes which remain poor in most patients. Recent observations have suggested the disturbances in the balance between ATP and adenosine may be integral to the vascular remodelling seen in PAH. CD39 is an enzyme important in regulating these nucleos(t)ides which may also provide a novel pathway to target for future therapies. This review summarises the role of adenosine signalling in the development and progression of PAH and highlights the therapeutic potential of CD39 for treatment of PAH.

## Introduction

Pulmonary arterial hypertension (PAH) is a progressive disorder characterised by pulmonary arterial vasoconstriction, vascular remodelling and smooth muscle cell proliferation. The resultant increase in pulmonary vascular resistance (PVR) leads to right ventricular afterload, hypertrophy and ultimately death due to right heart failure. Although the underlying pathogenesis of PAH is poorly understood, imbalances in prostacyclin, nitric oxide and endothelin-1 have been implicated, and many current therapies target these pathways [1]. Despite this, the prognosis for PAH remains poor with a 43% 5-year mortality [2] and as such the development of therapeutic strategies targeting novel pathways contributing to the pathobiology of PAH is of great importance.

## Pulmonary hypertension

PH is defined by a resting mean pulmonary arterial pressure (mPAP) greater than or equal to 20 mm Hg, measured by right heart catheterization [3]. PAH can be classified as pre- or post-capillary PH. Pre-capillary PAH is due to a primary elevation in the pressures in the pulmonary arterial system and post-capillary PH is due to elevations in the pulmonary arterial pressures that result from back pressure from the venous system or left side of the heart [3]. Nevertheless, the World Health Organization has largely standardised the PH classification into five groups based on the underlying aetiology [3]. Pulmonary hypertension is the umbrella under which all 5 categories sit, pulmonary arterial hypertension (PAH) is used to describe patients in group 1. Table 1 outlines these classification groups which aims to differentiate pulmonary arterial hypertension (PAH group 1) from secondary causes. Whilst these groups display similarities, they are distinct in terms of pathophysiology and clinical course.

**Table 1:** Updated clinical classification of pulmonary hypertension (PH) [3]

Group 1 – Pulmonary arterial hypertension
1.1 Idiopathic PAH 1.2 Heritable PAH 1.3 Drug- and toxin-induced PAH 1.4 PAH associated with: 1.4.1 Connective tissue disease 1.4.2 HIV infection 1.4.3 Portal hypertension 1.4.4 Congenital heart disease 1.4.5 Schistosomiasis 1.5 PAH long-term responders to calcium channel blockers (table 4) 1.6 PAH with overt features of venous/capillaries (PVOD/PCH) involvement (table 5) 1.7 Persistent PH of the newborn syndrome
Group 2 PH due to left heart disease
2.1 PH due to heart failure with preserved LVEF 2.2 PH due to heart failure with reduced LVEF 2.3 Valvular heart disease 2.4 Congenital/acquired cardiovascular conditions leading to post-capillary PH
Group 3 PH due to lung diseases and/or hypoxia
3.1 Obstructive lung disease 3.2 Restrictive lung disease 3.3 Other lung disease with mixed restrictive/obstructive pattern 3.4 Hypoxia without lung disease 3.5 Developmental lung disorders
Group 4 PH due to pulmonary artery obstruction
4.1 Chronic thromboembolic PH 4.2 Other pulmonary artery obstructions
Group 5 PH with unclear and/or multifactorial mechanisms
5.1 Haematological disorders 5.2 Systemic and metabolic disorders 5.3 Others 5.4 Complex congenital heart disease

*PAH*, pulmonary arterial hypertension; *PVOD*, pulmonary veno-occlusive disease; *PCH,* pulmonary capillary haemangiomatosis; *LVEF*, left ventricular ejection fraction

Group 1 is classified as those with pulmonary arterial hypertension (PAH) owing to a primary narrowing of the arterial vascular bed within the lungs. This narrowing can occur due to specific underlying causes such as familial traits, drugs or toxins, connective tissue diseases including scleroderma or lupus, infections such as human immunodeficiency virus (HIV) or schistosomiasis. If no clear underlying cause or association is found, it is termed idiopathic pulmonary arterial hypertension (iPAH) [3]. The remaining 4 groups are outlined in Table 1.

The symptoms of PAH, although initially very nonspecific, are generally progressive. They include breathlessness, fatigue, weakness, angina and syncope [4]. The nonspecific presentation along with poor correlation between severity and clinical signs often results in delays in diagnosis and advanced disease by the time the diagnosis is made [5, 6].

Currently PAH remains incurable and, for patients who are eligible, lung transplantation remains the only hope for long-term survival [7]. Current available therapies generally aim to dilate the small arterioles in the pulmonary vascular bed through vasodilatory medications or muscle relaxants [8–10]. Certainly, these advances in therapy often improve patients’ symptoms and quality of life; however, they do not improve long-term outcome [11]. Given none of the current therapies target the pulmonary vascular remodelling or inflammation which are now thought to underpin the pathogenesis, further understanding of these processes could potentially provide novel therapeutics. The quiescent pulmonary endothelium maintains an antithrombotic surface that facilitates the transit of plasma and cellular constituents throughout the pulmonary vasculature [12]. More recently, the importance of an intact endothelium in the homeostatic mechanisms that regulate vascular tone, cellular adhesion and blood fluidity have been better understood. It is now clear the intact and normally functioning endothelium has an integral role in maintaining an anti-inflammatory and antithrombotic micro-environment essential for the preservation of microvascular circulation and organ perfusion [13, 14]. Owing to its primary role, it is unsurprising that endothelial dysfunction results in a myriad of disease processes and endothelial dysregulation has been implicated in the pathogenesis of many occlusive vascular inflammatory vasculopathies such as pulmonary arterial hypertension [13–18]. PAH is emerging as a disease of endothelial dysfunction resulting in loss of normal vasodilator responses, abnormal wall remodelling and luminal narrowing of the pulmonary vessels [19].

Stiffening of the large elastic main pulmonary arteries is attributed to the lesions occurring in the distal pulmonary arteries ranging in diameter from 500 to 70um [20]. Three factors are thought to contribute to the increased pulmonary vascular resistance which characterise PAH: vasoconstriction, remodelling of the pulmonary vessel wall and thrombosis in situ [21]. Uncertainty remains surrounding the homology in pathogenesis across the different sub-types of PH. Nevertheless, the mechanisms driving PAH are still largely unclear despite advances in understanding the contributory factors such as inflammation, pulmonary endothelial cell dysfunction and aberrant cell proliferation in the vascular wall, as well as several gene mutations.

Purinergic signalling pathways are an essential regulator of the pulmonary vasculature and increasingly alterations in these pathways have been implicated in the pathogenesis of PAH with altered expression of ectonucleotidases, the integral regulators of purinergic signalling, described in patients with PAH [18, 22–24]. Intravascular nucleotide concentrations are regulated primarily by ectonucleotidases such as CD39 and CD73. To abrogate the pathological effects of extracellular ATP, a dynamic cascade of enzymes hydrolyses ATP to adenosine; CD39 hydrolyses the inflammatory ATP and prothrombotic ADP to AMP and subsequently CD73 dephosphorylates AMP into adenosine [25–27]. Extracellular nucleotide concentrations and adenosine homeostasis is largely governed by these ectoenzymes. CD39 appears to represent a built-in, molecular brake on endothelial and immune cells which ensures tight regulation of intravascular nucleotide concentrations, and thus vascular inflammation and thrombosis at sites of injury [28]. Owing to its role in short-term vascular tone and longer term control of cell proliferation, migration and death, it is unsurprising that purinergic signalling appears to play an important role in the development and progression of PAH.

## Endothelial dysfunction is paramount in the pathogenesis of PAH

Endothelial dysfunction is a term used to denote the phenotypic switch from the quiescent monolayer intent on preserving vascular fluidity to one that activates the host defence and promotes inflammation and thrombosis. It encompasses changes in the vasodilatory properties caused by reduction in the bioavailability of nitric oxide (NO) [29] and activation of molecular machinery which promote interactions between the endothelial layer, leukocytes and platelets such as increased expression of adhesion molecules E-selectin, ICAM-1 and VCAM-1 [12]. This imbalance impairs endothelial-dependent vasodilation but also results in a prothrombotic, pro-inflammatory and proliferative milieu which promotes vascular remodelling [30] in several diseases including atherosclerosis.

This is apparent in PAH where dysfunctional pulmonary endothelial cells contribute to the pulmonary vascular remodelling process as they foster proliferation and survival as well as migration of resident pulmonary vascular cells such as smooth muscle cells, myofibroblasts and pericytes [31, 32]. Chronically impaired production of vasoactive mediators, such as nitric oxide and prostacyclin, along with prolonged overexpression of vasoconstrictors such as endothelin-1, not only affect vascular tone and promote vascular remodelling but also foster a prothrombotic environment [20, 32–34].

Underpinning PAH is the subsequent local adapted release of particular chemokines, cytokines and growth factors which inhibit angiogenesis and drive pulmonary vascular remodelling. Endothelial cells from patients with PAH appear to have an abnormal phenotype with features such as decreased capacity for vascular tube formation, heightened aerobic glycolysis [34]. Furthermore, ECs from patients with PAH appear to lose some of their endothelial cell markers such a PECAM and CD31 and acquire mesenchymal cell markers. This pro-inflammatory phenotype is also characterised by surface expression of E-selectin, ICAM-1 and VCAM-1 [34] which promote interaction with inflammatory cells on the endothelial surface. Nevertheless, the exact mechanism responsible for the observed endothelial dysfunction in PAH remains unclear. Recent studies have focused on two possibilities—high shear stress and chronic hypoxia.

As pulmonary circulation is high-flow, laminar shear stress, defined as the tangential force per unit area caused by flowing blood, is imposed continuously upon the pulmonary endothelium. It has been shown to modulate the endothelial phenotype, including its barrier function [35, 36]. Shear forces play an intrinsic role in promoting quiescence or activation of the endothelial cell [37, 38] and EC response to shear force can be organ specific [37, 39]. Laminar shear stress can vary significantly between different organs and even throughout the vascular bed of an organ. This is apparent in the lung where in the microvascular shear forces may be 5–20 dyne/cm^2^ but between 10 and 50 dyne/cm^2^ in the large arteries [40] or much lower in the veins. Laminar shear stresses in PH can reach levels well above physiological state (> 80 dyne/cm^2^) [35]. In the pulmonary vasculature, conditions of increased shear stress result in loss off the normal EC cobblestone appearance and elongates the EC in the direction of the flow; failure to morphologically adapt is associated with a tendency to vascular remodelling [41–43]. Interestingly, ECs from microvascular pulmonary vessels of patients with PAH, but not those from proximal pulmonary arteries, exhibited this delayed morphological adaptation to high shear stress in vitro [41]. Additionally, pathological shear force has been shown to reduce vasodilator (i.e. NO and prostacyclin) release whilst also promoting vasoconstrictor (i.e. endothelin and thromboxane) release which appears to result in upregulation of endothelial alpha-smooth muscle actin without impacting on smooth muscle cell proliferation [35]. Shear stress alone, however, was insufficient to cause animals to develop PAH [43], suggesting maladaptive EC response to shear stress may be one of several “hits” required for the development and perpetuation of PAH.

Chronic hypoxia appears to be a potent driver of structural remodelling in the humans with PAH and experimental models. Within plexiform lesions, ECs appear to have disrupted hypoxia sensors, hypoxic-inducible factor (HIF1 and HIF) [44]. In a mouse model, disruption of the prolyl-4 hydroxylase 2 (PHD2) gene, the enzyme that facilitates degradation of HIF1 and HIF2, results in an obliterative pulmonary vascular remodelling and complex lesions resembling plexiform lesions found in human PAH [44]. Chronic hypoxia also appears to increase eATP concentrations with contributions from the endothelium and the circulating erythrocytes [22].

Furthermore, there may be a synergistic relationship between shear stress and hypoxia in stimulating eATP from EC [45].

Better understanding of the mechanisms that underpin EC adaptation in the context of shear stress, chronic hypoxia and endothelial toxins may provide opportunities for novel therapeutic targets. Crosstalk between damaged ECs and smooth muscle cells may exacerbate the pulmonary artery vasoconstriction driven by imbalance between endothelial-derived vasodilators such as NO and prostacyclin and vasoconstrictive factors such as endothelin-1. ATP release from damaged, hypoxic or shear-affected endothelial cells, and its subsequent downstream signalling via purinergic pathways, is potentially a significant player contributing to the development of pulmonary hypertension [22, 34].

## Purinergic signalling is disrupted in PAH

**Fig. 1:**
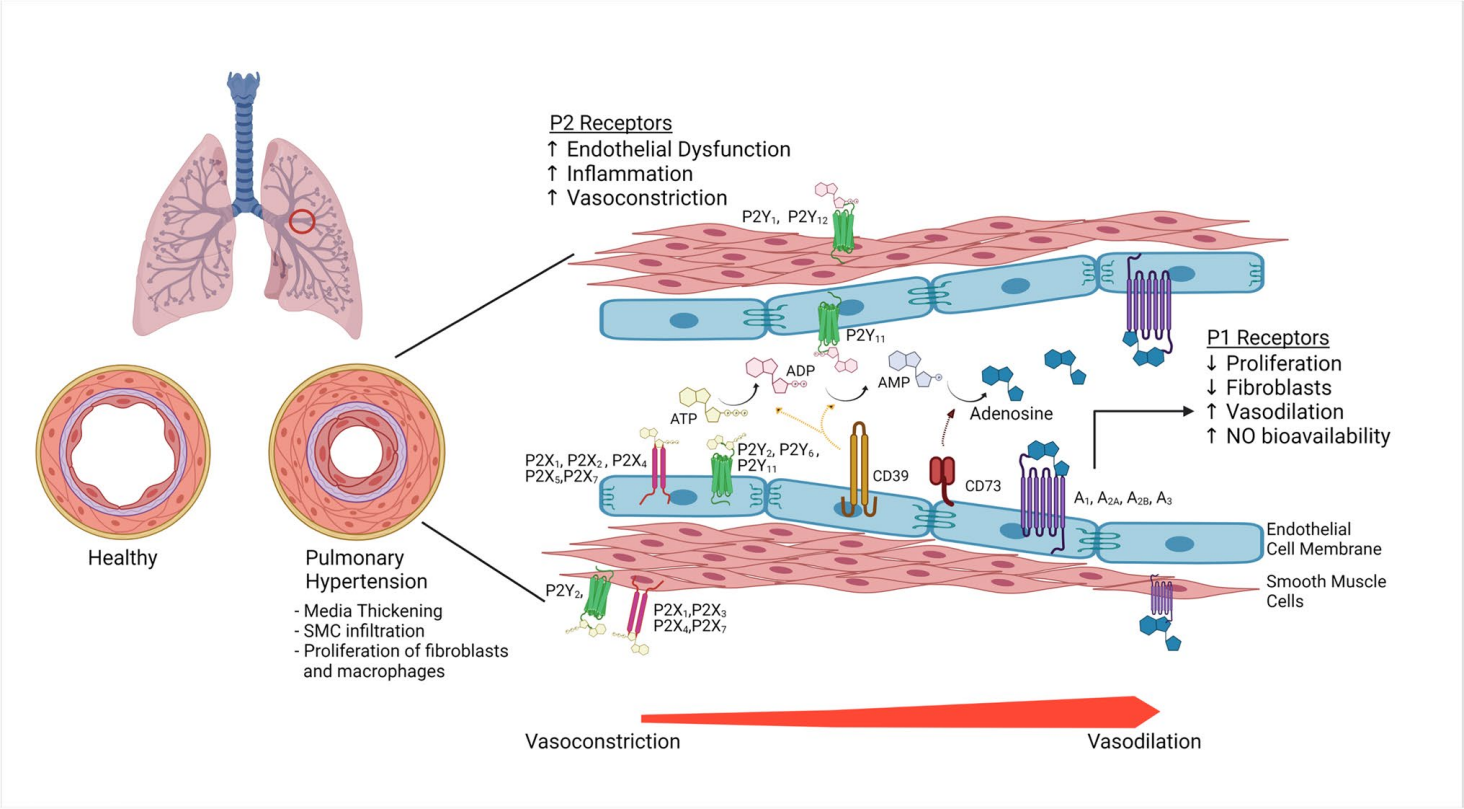
Pulmonary hypertension (PH) is characterised by vasoconstriction, vascular remodelling, and endothelial dysfunction. Characteristic findings include smooth muscle cell proliferation and infiltration, proliferation of fibroblasts, and increased inflammation in the adventia. Endothelial dysfunction promotes the release of extracellular ATP (eATP). CD39 is expressed on endothelial cells in the vasculature and converts ATP and ADP into AMP. AMP is then catabolised by CD73 to generate adenosine. Whilst ATP and ADP potentiate inflammation and vasoconstriction. The sub-types and distribution of P2 receptors which are activated by ATP and ADP are shown here throughout the endothelial cell (EC) and SMCs. Adenosine on the other hand promotes vasodilatation via the P1 receptors particularly A_2A_R.Whilst stimulation of the A_2B_R increases nitric oxide bioavailability, prolonged activation appears to promote vascular remodelling. (Created with BioRender.com)

Purinergic signalling plays an integral role in the maintenance of endothelial cell integrity and blood vessel patency; imbalance of the adenosine/ATP ratio is a potential contributor to the propagation of endothelial dysfunction in the development and progression of PAH (Fig. 1). The purinergic nucleotides adenosine triphosphate (ATP), adenosine diphosphate (ADP), adenosine monophosphate (AMP) and the nucleoside adenosine are extracellular signalling molecules that can signal downstream effector targets to modulate endothelial and smooth muscle cell growth, endothelial apoptosis, coagulation, vascular tone and inflammation [28, 46]. These ligands interact with a variety of cognate P1 (adenosine) and P2 (ATP and ADP) receptors to produce effects that may be complimentary or antagonistic to one another, depending upon tissue-specific receptor sub-types and concentrations [47, 48]. Nucleot(s)ides act via a series of purinergic receptors, of which there are 2 subfamilies, purinergic receptor 1 (P1R) and purinergic receptor 2 (P2R). The P1R family, also known as adenosine receptors, are a group of G-protein coupled receptors including A1R, A_2A_R, A_2B_R and A3R. The P2R subfamily has 2 subgroups consisting of 7 P2XRs (P2X_1-7_R) and 8 P2YRs (P2Y_1_R, P2Y_2_R, P2Y_4_R, P2Y_6_R, P2Y_11_R, P2Y_12_R, P2Y_13_R and P2Y_14_R), and these can be stimulated by ATP, ADP or UTP [49]. Whilst there is heterogeneity in the expression of these receptors across different animal species, all P1 receptors can be found in the lung tissue of humans and mice [50–52]. Additionally, P2X_1_, P2X_2_, P2X_4_, P2X_5_, P2X_7_, P2Y_2_, P2Y_6_, P2Y_11_ have all be demonstrated to be present on the pulmonary endothelium, whereas P2X_1_, P2X_3_, P2X4, P2X_7_, P2Y_1_, P2Y_12_ are expressed on the smooth muscle cells in the pulmonary vasculature [53–57].

ATP is released into the extracellular space in response to tissue damage or cellular stress and acts as danger-associated molecular pattern (DAMP) and binds to P2 receptors to prompt signalling cascades to induce an inflammatory response [58]. Both shear stress and chronic hypoxia induce release of ATP from the endothelial surface [45, 59, 60]. Additionally, ATP is released from erythrocytes is states of hypoxia which is increasingly recognised as a critical regulator in tissue perfusion [61]. The role of eATP in the pulmonary vascular bed is variably dependent on the receptor and cell on which it is exerting its activity. ATP causes vasoconstriction by activation of the P2X_1_ receptor on smooth muscle cells [62] but can also act via the P2Y_1_ and P2Y_2_ receptors to release NO resulting in vessel relaxation [59, 63]. Shear stress promotes the release of eATP by pulmonary endothelial cells. Both flow-induced shear stress and eATP are activators of transient receptor potential vanilloid 4 (TRPV4) channels in pulmonary arteries. More recently, it has been shown that this is likely mediated via eATP-potentiation of the endothelial TRPV4 which results in calcium dependent upregulation of eNOS to dilate pulmonary arterioles [64, 65]. Impaired caveolin-1-TRPV4 signalling has been shown to reduce endothelial vasodilatation and consequently increase pulmonary artery pressure, which may be a key pathological event in development of PAH [64, 65]. Intriguingly, hypoxia in both humans and mice also appears to induce upregulation of the P2X_1_ receptor in the lungs in a maladaptive response–promoting vessel vasoconstriction [22]. ADP promotes platelet activation and thrombosis but also can exert vasoconstrictive activity via P2Y_1_ and P2Y_12_ particularly in the context of hypoxia [54].

Adenosine on the other hand is a potent immune-suppressor particularly of cells that express A_2_ and A_3_ receptors, such as lymphocytes [66]. Adenosine is an important bioactive agent in states of vascular inflammation; its effects are mediated on both vascular cells and leukocytes [67]. In addition, adenosine has known antithrombotic effects by blocking induction of tissue factor via A_2A_ and A_3_ receptors [68, 69], particularly during ischemic or atherosclerotic processes, and it also modulates the expression of antiapoptotic genes and has anti-inflammatory properties [69]. The pulmonary vasculature is one of the few vascular beds where the P1 receptors exert dual function promoting both vascular contraction and relaxation to maintain basal tone [70]. In the pulmonary vasculature adenosine stimulation of the A_1_ receptor, to which it has high affinity, results in vasoconstriction. However, stimulation of the A_2A_ generally results in vasodilation. In the low adenosine state seen in patients with PAH [23], the variable affinity adenosine has with its G-protein-coupled receptors may play a key role in the prevailing phenotype of vasoconstriction due to its propensity for the A_1_ receptor over others.

Indeed, plasma adenosine concentrations from patients with PAH are lower than in healthy subjects [23]. This finding is corroborated in a study of newborn lambs with hypoxia-induced PAH where lower adenosine levels were noted compared to normoxic lambs [71]. As such, adenosine has long been of interest in the therapeutic strategy in PAH owing to its vasodilatory activity. Reduction in right ventricular pressure was seen with infusion of adenosine in newborn lambs; this effect was apparent at lower doses in hypoxic lambs suggesting that hypoxia may sensitise the pulmonary vasculature to adenosine [71]. Curiously, response to adenosine in patients appears to be dependent on the underlying cause of the PH. In patients with elevated pulmonary pressures immediately post cardiac surgery intravenous administration adenosine-reduced PAP and improved cardiac output at doses which did no compromise systemic blood pressure [72]. Therapeutic efficacy has also been seen in patients with PH associated with congenital heart disease [73] and prematurity in the neonate [74, 75]. Nevertheless, only around 10–20% of patients with idiopathic PAH had a reduction in their PAP with adenosine infusion [76, 77]. The utility of adenosine as a therapeutic strategy has been hampered by its exceptionally short half-life (5–10 s), and its propensity for systemic effects including hypotension with accompanying increased heart rate and cardiac output. As such, the role of more specific purinergic signalling pathways continues to be explored.

## A_2A_R stimulation is appears to be protective in pulmonary hypertension

A_2A_R is an adenosine receptor, located on the endothelium and the smooth muscle cells, whose activation leads to vasodilation [78, 79]. The role of A_2A_R in the development of PAH has been explored in several animal models including an A_2A_R genetic knockout (KO) mouse. The genetic inactivation of A_2A_R selectively and spontaneously produced PAH with associated increased RVS pressures, right ventricular hypertrophy as well as associated increased smooth muscle proliferation and collagen deposition [80].

Lack of A_2A_R signalling appears to correlate with increased mRNA protein expression of the Ras homolog gene family member A and Rho kinase (ROCK)1 particularly on pulmonary endothelial and smooth muscle cells [80, 81]. Activation of Rho A and its downstream effectors ROCKs activate Ca^2+^/calmodulin-dependent muscle contraction but also appear to inactivate Ca^2+^-independent smooth muscle relaxation. In a chronic hypoxia induced model of PH in rats, inhalation of fasudil, a Rho kinase inhibitor, markedly reduced the development of PH and improved lung vascular remodelling [82].

A_2A_R agonism appears to be protective in models of PAH. LASSBio-1359 and LASSBio-1386, both strong A_2A_R agonists, have been shown to have a potent vasodilator effect monocrotaline-induced PAH in rats [83, 84]. Treated animals showed reduced PAP as well as reduced vessel wall hypertrophy with chronic administration. This finding was further attributed to A_2A_R agonism, since the vascular effects of LASSBio-1359 and LASSBio-1386, could be reduced by using a selective A_2A_R antagonist [83, 84].

Taken together, these observations suggest that the low adenosine state seen in PAH and consequential reduction in A_2A_R activation results in Rho/ROCK-driven vasoconstriction and vascular cell hyperproliferation. Owing to its anti-vasoconstrictive and anti-remodelling properties, stimulation of A_2A_R signalling is a potential therapeutic option in PAH.

## The role of A_2B_ is uncertain in pulmonary hypertension

Unlike A_2A_R signalling, which may be protective in the development of PAH, the A_2B_R is known to promote the development of PAH. It is the receptor with the lowest affinity for adenosine [85] and whilst stimulation of the A_2B_R was generally thought to promote pulmonary vasodilation [86, 87], it has emerged as the likely modulator of pulmonary vascular remodelling with prolonged activation [88, 89].

A_2B_R appears to be upregulated on the pulmonary vasculature in various forms of PH, however, particularly those related to interstitial lung disease [89, 90], chronic obstructive airways disease [88] and idiopathic PAH [89]. Nullification of A_2B_R signalling, either using pharmacological inhibition with GS-6201 or genetic deletion of A_2B_R, appears to be protective in animal models of bleomycin-induced and lung-injury induced PAH with reduction in the vascular remodelling seen in these animals [88, 91].

Interestingly, direct A_2B_R activation promoted interleukin-6 and endothelin-1 release from both SMCs and ECs and cell culture medium from A_2B_R-stimulated ECs promoted proliferation in SMCs [91]. This could be abrogated using an A_2B_R blockade on SMCs where the release of several remodelling mediators such as interleukin 6 (IL-6) and hyaluronan synthase 2 were reduced [89]. Unsurprisingly, specific deletion of A_2B_R on the SMC of mice protected them from the development of PH and abolished the vascular remodelling seen in bleomycin-induced PH [89]. Furthermore, conditional deletion of A_2B_R in myeloid cells in a mouse model of lung injury–induced PH altered the inflammatory milieu. Animals had less IL-6 in bronchoalveolar lavage fluid and reduced pulmonary fibrosis [92].

Despite the findings regarding the role of A_2B_R, long -term subcutaneous infusion of adenosine or NECA, a non-selective P1R agonist, abrogates proliferation of the vascular cells and subsequent vascular remodelling which underpins PH development in chronic hypoxia [52]. Some hypothesise that under acute conditions, hyperactivation of the A_2B_R by adenosine is protective and leads to lung tissue repair [91]. Nevertheless, in humans with lung fibrosis, sustained activation of this receptor appears to be deleterious contributing directly to development of pulmonary vascular remodelling and P(A)H [90].

## CD39 is the molecular break regulating extracellular nucleot(s)ide concentrations

Intravascular nucleotide concentrations are regulated primarily by the ectonucleotidase CD39 [ectonucleoside triphosphate diphosphohydrolase 1 (ENTPD1) and CD73 (5-nucleotidase). Extracellular ATP functions as a danger signal (DAMP), triggering activation of P2 receptors and downstream pro-inflammatory responses [93].

In addition to its role in promoting adenosine generation, CD39 appears to be integral in the leukocyte trafficking response across the endothelial surface in response to chemotactic stimuli [28, 94]. Via rapid alterations in the purine concentration in the proximity of endothelial or immune cells, CD39 regulates immune cell adhesion to the endothelial layer [95]. Immune cell adhesion is generally promoted by an ATP-rich environment and inhibited by adenosine [94, 95]. Mouse models lacking CD39 display increased leukocyte adhesion to the vascular endothelium [96–98]. Impaired adenosine generation in these animals resulted in increased endothelial cell activation, greater monocyte recruitment and platelet aggregation and increased endothelial permeability suggesting a critical role for CD39 in the pathophysiology of vascular inflammation and microthrombosis [96–100].

Crucially, modulation of CD39 has been demonstrated to have therapeutic benefit in several diseases underpinned by endothelial dysregulation and microthrombosis. In a mouse model of myocardial infarction, CD39 deficiency resulted in increased susceptibility to myocardial injury [101]. Conversely, overexpression of CD39-induced protection from myocardial infarction as measured by infarct size in both mouse [102] and pig [103] models of cardiac ischemia. CD39 overexpression has been found to mitigate stroke [99], hypertension in pre-eclampsia [104] and antiphospholipid-related miscarriage [105].

## CD39 expression is downregulated in PAH

Whilst purinergic signalling is a potent modulator of pulmonary vascular homeostasis, this fine balance is achieved through the activity of ectonucleotidases. Changes in the CD39/CD73 axis have recently been hypothesised to cause the imbalance in the extracellular ATP/adenosine ratios playing a central role in the pathophysiology of PAH [22].

Elevated levels of functional CD39 were detected on microparticles from patients with idiopathic PAH when compared with healthy patients [18]. The significance of this finding was unexpected and difficult to interpret, and study authors concluded that additional work was needed to elucidate whether CD39 could be implicated in the pathogenesis of PAH or as a compensatory response. Since then, studies have consistently shown reduced CD39 expression on pulmonary vascular ECs from patients with idiopathic PAH compared with healthy controls [22, 56] and interestingly, CD39 expression is reduced in vessels with more severe vessel remodelling [6]. Cultured ECs lacking CD39 appear to have an apoptotic-resistant phenotype [56] and an ATP-rich environment has been shown to promote pulmonary smooth muscle migration and proliferation [22]. Poor CD39 expression in the small pulmonary vessels ostensibly may be the underlying cause of the adenosine poor state seen in PAH.

The role of CD39 and its downstream impacts in PAH have been further explored using several animal models. In a chronic hypoxia model of PAH, CD39-knockout mice were found to have significantly elevated ATP:adenosine ratios and went on to develop an unexpectedly severe phenotype of pulmonary hypertension [22]. This phenotype could be salvaged with reconstitution of functional CD39 using soluble apyrase (an endoculeotidase with ATPase and ADPase activity) or blockade of the P2X_1_ receptor, the predominant ATP receptor [22]. The activity of CD39 can be potentiated using apelin, an endogenous peptide which binds its respective G-protein coupled receptor, in both cultured pulmonary endothelial cells from patients with PAH as well as those isolated from monocrotaline-induced PH in rats [56]. Consistent with this finding, apelin has been demonstrated to abrogate the effects of PAH in both animals and patients [106, 107]. Together, this suggests that the therapeutic benefit derived from apelin is at least in some part mediated by modulation of downstream purinergic signalling in an otherwise CD39-impovrished environment. Inarguably, the downregulation of CD39 on pulmonary endothelial cells may alter the delicate ATP:adenosine ratios promoting vasoconstriction and vascular remodelling. Reconstitution of CD39 mediated purinergic signalling on the pulmonary endothelial surface may provide an avenue for novel therapeutic targets in the future.

## Conclusions

Over the last 20 years, there has been advances in our understanding of the role of adenosine and its regulators in PAH which may provide a novel therapeutic target. This is a multifactorial issue exacerbated by maladaptive endothelial responses to altered conditions of shear stress and hypoxia. Sustained abnormal CD39 activity in the pulmonary vasculature results in ATP accumulation and adenosine diminution promoting the vasoconstriction and vascular remodelling seen in PAH. However, the complex interplay between the four P1R and their selective agonism or antagonism requires further investigation if clinical efficacy is to be achieved.
